# What the papers say

**DOI:** 10.1093/jhps/hnae005

**Published:** 2024-04-09

**Authors:** Ali Bajwa

**Affiliations:** Villar Bajwa Practice, Princess Grace Hospital, London W1G6PU, UK

The *Journal of Hip Preservation Surgery* (*JHPS*) is not the only place where work in the field of hip preservation can be published. Although our aim is to offer the best of the best, we are continually fascinated by work, which finds its way into journals other than our own. There is much to learn from it, and so *JHPS* has selected six recent and topical subjects for those who seek a summary of what is taking place in our ever-fascinating world of hip preservation. What you see here are the mildly edited abstracts of the original articles, to give them what *JHPS* hopes is a more readable feel. If you are pushed for time, what follows should take you no more than 10 min to read. So here goes …

## THE AUTOLOGOUS CHONDRAL PLATELET-RICH PLASMA MATRIX IMPLANTATION. A NEW THERAPY IN CARTILAGE REPAIR AND REGENERATION: MACROSCOPIC AND BIOMECHANICAL STUDY IN AN EXPERIMENTAL SHEEP MODEL

The authors from Spain [1] carrying out this animal study state that the articular cartilage injuries are a severe problem, and the treatments for these injuries are complex. The present study investigates a treatment for full-thickness cartilage defects called Autologous Chondral Platelet-Rich Plasma Matrix Implantation (PACI) in a sheep model.

Chondral defects 8 mm in diameter were surgically induced in the medial femoral condyles of both stifles in eight healthy sheep. Right stifles were treated with PACI and an intraarticular injection with a plasma rich in growth factors (PRGF) solution [treatment group (TRT)], while an intraarticular injection of Ringer’s lactate solution was administered in left stifles [Control group (CT)]. The limbs’ function was objectively assessed with a force platform to obtain the symmetry index, comparing both groups. After 9 and 18 months, the lesions were macroscopically evaluated using the International Cartilage Repair Society and Goebel scales.

Regarding the symmetry index, the TRT group obtained results similar to those of healthy limbs at 9 and 18 months after treatment. Regarding the macroscopic assessment, the values obtained by the TRT group were very close to those of normal cartilage and superior to those obtained by the CT group at 9 months.

The authors note that this new bioregenerative treatment modality can regenerate hyaline articular cartilage. High functional outcomes have been reported, together with a good quality repair tissue in sheep. Therefore, they concluded that PACI treatment might be a good therapeutic option for full-thickness chondral lesions.

## DEFINING HIP CARTILAGE REPAIR: A MODIFIED DELPHI STUDY TO ESTABLISH THE MAGNETIC RESONANCE EVALUATION OF THE REPAIR OF CARTILAGE IN THE HIP (MERCH) SCORE

In this study, the authors from Canada [2] aim to develop a standardized scoring system to evaluate pre- to post-operative repair or reconstruction of hip cartilage using magnetic resonance imaging (MRI). A two-phase modified Delphi study was conducted. Phase 1 involved a survey with suggested criteria and diagrams to define various stages of articular cartilage repair and Phase 2 involved an expert consensus meeting that discussed the survey responses and voted on final scoring criteria. The survey was emailed to members of the Canadian Hip Preservation Research Collaborative (CHIPR) and respondents included both board certified orthopedic surgeons as well as musculoskeletal radiologists.

Overall, there were 17 survey respondents from Canada and most (47%, 8/17) participants agreed that the ‘minimum’ MRI protocol needed to evaluate cartilage repair was a 3.0T MRI and 94% (17/18) agreed that the minimum time post-operatively that they felt they would be able to accurately evaluate cartilage repair on an MRI was 12 months. Following Phases 1 and 2, the final Magnetic Resonance Evaluation of the Repair of Cartilage in the Hip (MERCH) score was developed with seven domains, three criteria per domain: (i) volume fill of cartilage defect, (ii) integration into adjacent cartilage, (iii) surface of the repair tissue, (iv) structure of the repair tissue, (v) bony overgrowth, (vi) subchondral changes and (vii) delamination. The score ranges from 60 (optimal) to −20 points (worst/none).

This consensus project established a new MRI scoring system to evaluate post-operative cartilage restoration of the hip. The authors felt that the implementation of the MERCH score was essential in their ability to guide patient management and expectations in a rapidly evolving field and would help with standardizing their evaluation of cartilage repair in future research trials.

## ULTRA-DURABLE CELL-FREE BIOACTIVE HYDROGEL WITH FAST SHAPE MEMORY AND ON-DEMAND DRUG RELEASE FOR CARTILAGE REGENERATION

The authors from China [3] point out that osteoarthritis is a worldwide prevalent disease that imposes a significant socioeconomic burden on individuals and healthcare systems. Achieving cartilage regeneration in patients with osteoarthritis remains a challenge in clinical setting. In this work, they construct a multiple hydrogen-bond crosslinked hydrogel loaded with tannic acid and Kartogenin by polyaddition reaction as a cell-free scaffold for *in vivo* cartilage regeneration, which featured ultra-durable mechanical properties and stage-dependent drug release behavior. They demonstrated that the hydrogel could withstand 28 000 loading–unloading mechanical cycles and exhibited fast shape memory at body temperature (30 s) with the potential for minimally invasive surgery. The authors found that the hydrogel could also alleviate the inflammatory reaction and regulate oxidative stress *in situ* to establish a microenvironment conducive to healing. They showed that the sequential release of tannic acid and Kartogenin could promote the migration of bone marrow mesenchymal stem cells into the hydrogel scaffold, followed by the induction of chondrocyte differentiation, thus leading to full-thickness cartilage regeneration *in vivo*. Authors felt that their work might provide a promising solution to address the problem of cartilage regeneration.

## CLINICAL OUTCOMES AFTER OPEN AND ENDOSCOPIC REPAIR OF PROXIMAL HAMSTRING TENDON TEARS AT A MINIMUM FOLLOW-UP OF 5 YEARS

The authors from USA **[4]** note that the current evidence supports favorable short-term clinical outcomes with few complications after surgical management of proximal hamstring injuries; however, the durability of clinical benefits beyond approximately 2 years after surgery is unknown.

The aim of their study was to evaluate patient-reported clinical outcomes and complication rates associated with open and endoscopic repair of proximal hamstring tears at minimum 5 year follow-up in this case series.

A single-surgeon registry of patients was queried between 1 October 2014 and 31 December 2017 to identify patients who underwent open or endoscopic repair of a proximal hamstring tear. Patients who reported minimum 5 year follow-up data were included. Multiple patient-reported outcome measures, including the Hip Outcome Score Activities of Daily Living (HOS-ADL) and Sports-Specific (HOS-SS) subscales, 12-Item International Hip Outcome Tool (iHOT-12) and Patient-Reported Outcomes Measurement Information System (PROMIS) Physical Function (PF) and Pain domains, along with surgical complications, were analyzed.

Among 35 eligible patients (65.7% female; mean age, 52.3 years), 24 had full-thickness tears and 11 had partial-thickness tears. There were 23 open repairs and 12 endoscopic repairs. Mean duration from symptom onset to surgical intervention was 37.9 weeks. At a mean follow-up of 69.0 months, mean post-operative outcome scores were as follows: HOS-ADL, 86.8 ± 12.7; HOS-SS, 83.1 ± 19.5; iHOT-12, 86.3 ± 14.9; PROMIS-PF, 50.0 ± 11.8; and PROMIS–Pain, 50.2 ± 7.9. Regarding complications, 28.6% of patients had a complication including persistent peri-incisional numbness (11.4%), wound infection (11.4%), post-operative neuropathy (8.6%), and revision surgery (2.9%).

They concluded that both open and endoscopic surgical techniques for repair of proximal hamstring injuries produced favorable patient-reported clinical outcomes at a minimum 5 year follow-up.

## PATIENT OUTCOMES ARE NOT IMPROVED BY PLATELET-RICH PLASMA INJECTION ONTO THE CAPSULE AT THE TIME OF CLOSURE DURING HIP ARTHROSCOPY FOR FEMOROACETABULAR IMPINGEMENT SYNDROME

In this study, Morris et al. [5] investigate the effect of platelet-rich plasma (PRP) injection onto the capsule at the time of closure on outcomes of patients undergoing hip arthroscopy for femoroacetabular impingement syndrome.

Patients who underwent hip arthroscopy between January 2014 and December 2021 were retrospectively identified. The first cohort included patients who received PRP injection onto the capsule following capsular closure at the conclusion of the case. The second cohort did not receive PRP. Pain scores on a visual analog scale, Modified Harris Hip Scores, Single Assessment Numeric Evaluation (SANE), as well as Patient-Reported Outcomes Measurement Information System Physical Function scores were obtained preoperatively as well as at multiple time points post-operatively up to 2 years.

In total, 345 patients were included in the study, with 293 in the PRP cohort and 52 in the non-PRP cohort. There was no significance difference in age, sex or preoperative pain and patient-reported outcome scores between the two groups (modified Harris Hip Score, *P* = 0.38; Patient-Reported Outcomes Measurement Information System Physical Function, *P* = 0.48), except for preoperative SANE scores, which had a greater baseline in the PRP group. Using both observed data as well as repeated measure analysis of variance model to estimate for missing data after baseline, we found there were no differences in visual analog scale pain scores or patient-reported outcome scores at any time point. There was similarly no difference in change from baseline for SANE scores. There was no difference in rate of revision surgery between the two cohorts.

The authors thus concluded that, based on the results of this study, intraoperative PRP injection onto the capsule at the time of capsular closure does not improve outcomes of patients undergoing hip arthroscopy for femoroacetabular impingement syndrome.

## HIP MRI IN FLEXION ABDUCTION EXTERNAL ROTATION FOR ASSESSMENT OF THE ISCHIOFEMORAL INTERVAL IN PATIENTS WITH HIP PAIN—A FEASIBILITY STUDY

The authors from Switzerland [6] assess the feasibility of flexion-abduction-external rotation (FABER) magnetic resonance imaging (MRI) of the hip to visualize changes in the ischiofemoral interval and ability to provoke foveal excursion over the acetabular rim.

In this IRB-approved retrospective single-center study, patients underwent non-contrast 1.5T hip MRI in the neutral and FABER position. Two readers measured the ischiofemoral interval at three levels: proximal/distal intertrochanteric distance and ischiofemoral space. Subgroup analysis was performed for hips with/without high femoral torsion, or quadratus femoris muscle edema (QFME), respectively. A receiver operating curve with calculation of the area under the curve (AUC) for the prediction of QFME was calculated. The presence of foveal excursion in both positions was assessed.

One hundred ten patients (121 hips, mean age 34 years, 67 females) were evaluated. FABER-MRI led to narrowing of the ischiofemoral interval which decreased more at the proximal (mean decrease by 26 ± 7 mm) than at the distal (6 ± 7 mm) intertrochanteric ridge. With high femoral torsion/ QFME, the ischiofemoral interval was significantly narrower at all three measurement locations compared with normal torsion/no QFME. Accuracy for predicting QFME was high with an AUC of 0.89 (95% CI 0.82–0.94) using a threshold of ≤7 mm for the proximal intertrochanteric distance. With FABER-MRI, foveal excursion was more frequent in hips with QFME (63% versus 25%).

The authors concluded that hip MRI in the FABER position is feasible, visualises narrowing of the ischiofemoral interval and can provoke foveal excursion.
